# Postoperative Exostosis: Ectopic Ossification After Pericardial Window Procedure

**DOI:** 10.1016/j.atssr.2023.06.004

**Published:** 2023-06-28

**Authors:** Ahmed Mostafa, Mohamad Alhalabieh, Dimitrios Xourafas, Tiffany Schatz

**Affiliations:** 1 Department of General Surgery, Nazareth Hospital, Trinity Health Mid-Atlantic, Philadelphia, Pennsylvania

## Abstract

A 58-year-old Hispanic man had persistent epigastric pain after pericardial window procedure for viral pericarditis several years earlier. After review of a computed tomography scan, a calcified epigastric subcutaneous mass was detected. The patient elected to undergo surgical excision of the mass, which revealed a heterotopic ossified lesion arising from the inferior margin of the xiphoid process. The patient reported marked clinical improvement after the operation that has been sustained postoperatively. This case highlights the importance of considering heterotopic ossification as a potential cause of persistent pain after pericardial window procedure.

This case chronicles a 58-year-old Hispanic man who underwent a subxiphoidal pericardial window for a pericardial effusion in 2019. The cause of the effusion was viral pneumonia complicated by acute pericarditis. Since the procedure, he was seen by several specialists and had multiple emergency department visits for epigastric pain, yielding no significant findings.

The patient presented to our thoracic surgery clinic on August 3, 2022, complaining of subxiphoid pain severely compromising his quality of life. He described the pain as a dull ache that persists throughout the day and worsens with movement. The pain has been present for several months and is not relieved by over-the-counter pain medications. Review of a computed tomography scan of the chest, abdomen, and pelvis revealed an ossified lesion extending from the xiphoid process (Figure 1). After discussion, it was decided that surgical excision of the lesion was the best course of action. On August 10, 2022, we proceeded to the operating room, acknowledging that the pain may be unrelated to the previous pericardial window preformed and that reoperation may aggravate his current condition.

**Figure 1:**
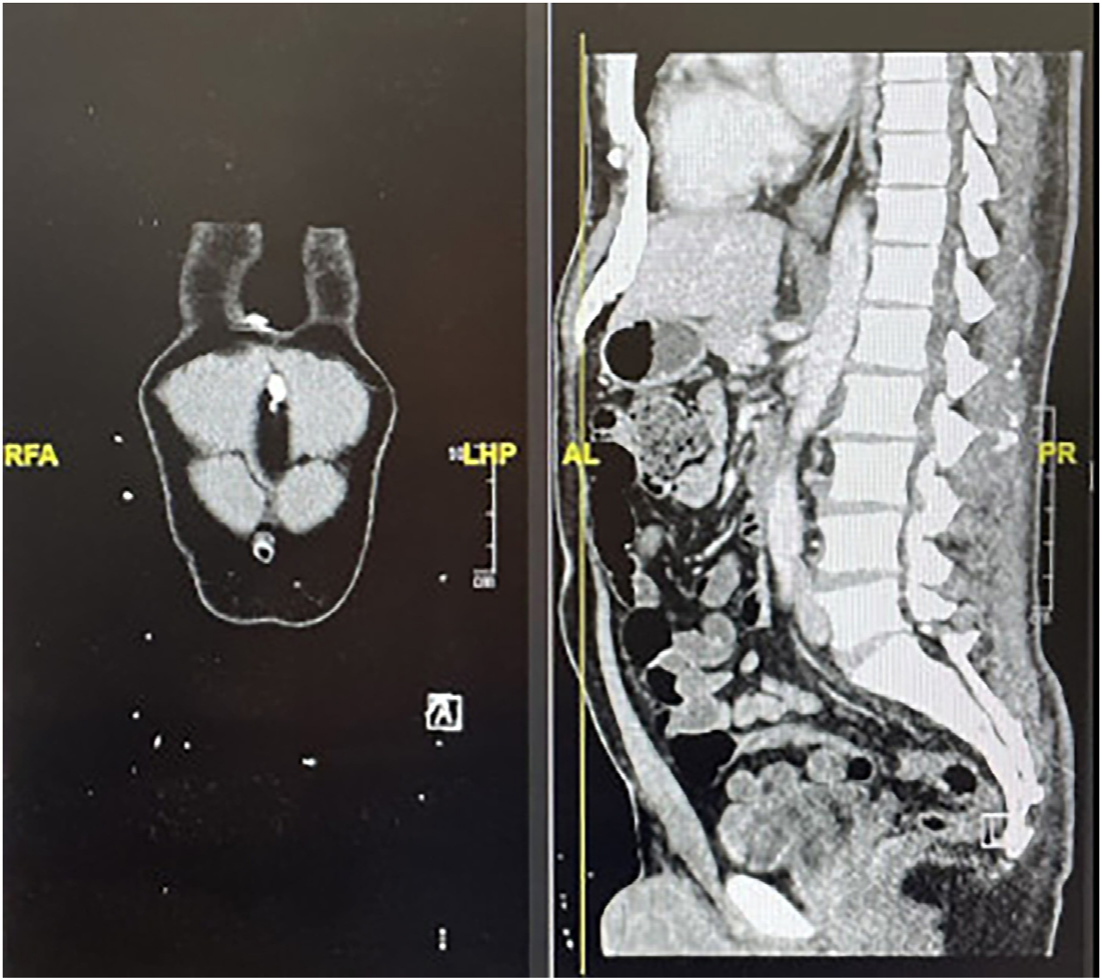
Preoperative computed tomography scan. (AL, anterior left; LHP, left head posterior; PR, posterior right; RFA, right foot anterior.)

In the operating room, a vertical midline 10-cm incision incorporating the old scar was made. Subcutaneous tissues were carefully dissected down to an ossified fibrous scar. The scar was freed from surrounding fascia and muscle in its entire length. After several failed attempts at transecting the mass with a No. 10 scalpel blade and electrocautery, a bone cutter was used, and the mass was successfully removed. It measured 3 × 5 cm (Figure 2, Figure 3) in maximum diameter. Afterward, 50 mg of diluted ketamine solution was injected into the cavity (Figure 2) before deep dermal and skin closure. Ketamine promotes local inflammatory homeostasis without affecting local wound healing.^1^^,^^2^ In the postanesthesia care unit, the patient reported marked clinical improvement with appropriate postoperative incisional pain. We recommended close follow-up to discuss the results of the pathologic examination and to reassess clinical progression. Pathologic examination revealed 5.5 × 3 × 2.8-cm tan-brown bone tissue with unremarkable marrow elements. He remains objectively pain free since surgery.

**Figure 2:**
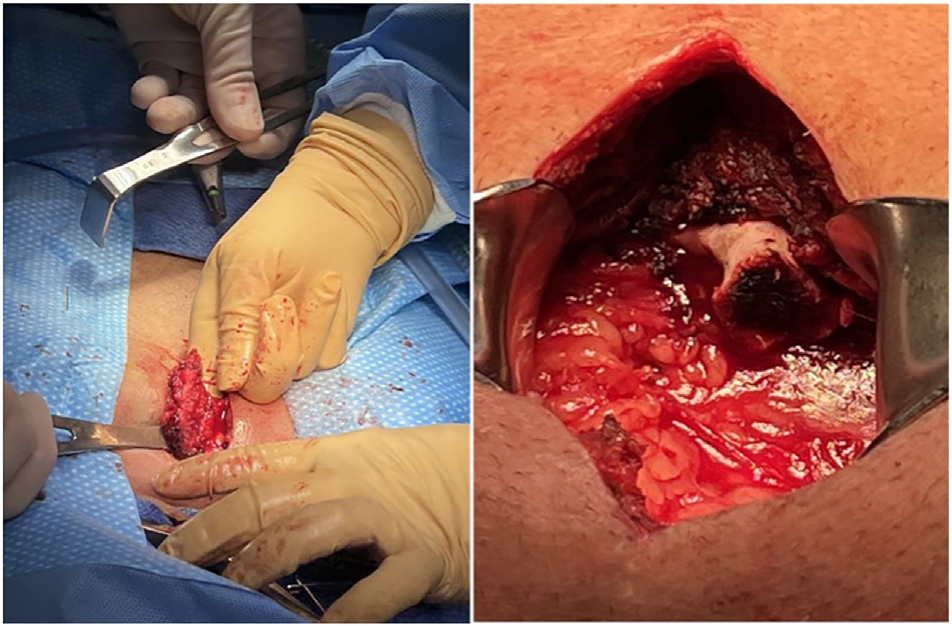
Specimen in situ and postresection bed.

**Figure 3:**
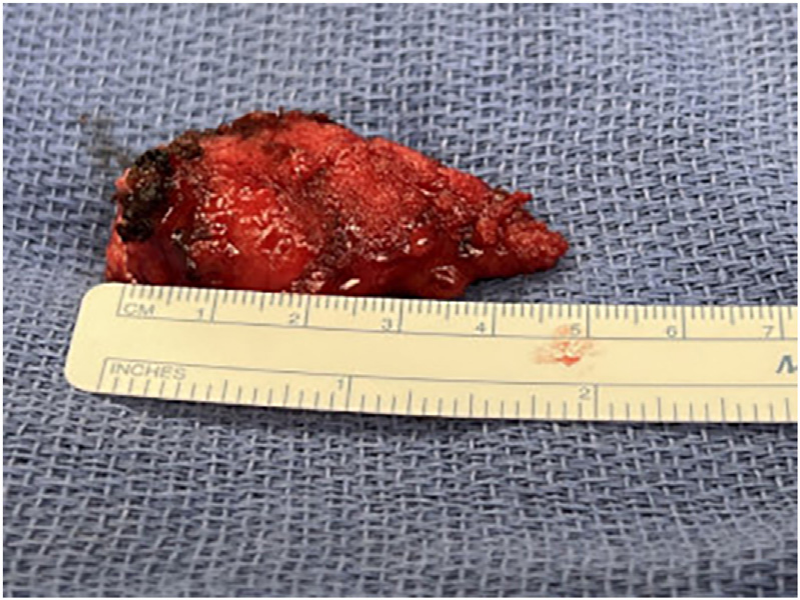
Excised ossified lesion measuring 5.5 × 3 × 2.8 cm.

The patient was seen in the clinic on postoperative day 14 and reported complete resolution of epigastric pain and tenderness. He stated that he was able to perform his daily activities without limitations. He was appreciative for having the lesion resected.

## Comment

Nonhereditary heterotopic calcifications were first documented in 1901 by Askanazy with a case of myositis ossificans traumatica. The condition is common in the athletic population after major or repeated trauma. It is theorized that in the center of traumatized tissue, under anoxic conditions, pluripotent mesenchymal cells can develop. An inflammatory background of prostaglandin E_2_, bone morphogenic protein 4, and stromal cell–derived factor 1 induces osteogenesis. Osteoinduction is mediated by local, neural, or humoral factors.

Neurogenic heterotopic calcification is linked to traumatic brain and spinal injuries, mainly affecting proximal periarticular muscles in paretic limbs with increased spasticity. Elevated levels of humoral factors, such as transforming growth factor β, insulin-like growth factor 2, platelet-derived growth factor, interleukin 1, and interleukin 6, are seen in the serum of patients with traumatic brain injury and have been shown to increase the activity of osteoblastic cells in skeletal muscle cultures.^3^

Midline thoracic and abdominal incisions carry a 25% risk of such calcifications. Two main principal theories are sustained. The first is the seeding of periosteal or perichondral tissue, probably from the xiphoid process or symphysis pubis into the nearby fascial planes or subcutaneous tissues. Even without direct damage or resection, seeding is suggested. The second theory relies on the fertile ground of pluripotent cells that under the right conditions can differentiate into osteoblasts or chondroblasts.^4^ Postoperative ossification commonly occurs with vertical surgical incisions, either midline or paramedian. To date, no documented cases of postoperative ossifications after horizontal surgical incisions exist. Osteogenic calcification predominantly occurs in men ranging in age between 50 and 75 years. There is no correlation between closure techniques and suture material used.

Radiotherapy and nonsteroidal anti-inflammatory drugs have been used to prevent formation and recurrence of calcification after surgical excision. These therapeutic modalities arrest the rapidly dividing pluripotent cells and curb the osseous inflammatory response, respectively. Bisphosphonates are also used to prevent bone mineralization, but calcification recurs when therapy is withdrawn.

In conclusion, nonhereditary heterotopic calcifications can range from asymptomatic subcutaneous masses to debilitating periarticular or thoracoabdominal lesions. Essentially, 3 conditions are required: an eliciting event, mesenchymal cells, and an inductive environment. Local occurrences, such as limb injuries or surgical operations, or generalized occurrences, such as catastrophic brain or spinal damage, initiate the process. Nearby dormant mesenchymal cells revert to pluripotent cells that are influenced by local, neural, or humoral factors to undergo osteogenesis. Understanding high-risk populations can allow preventive measures, such as preemptive nonsteroidal anti-inflammatory drug treatment in hip arthroplasty patients and radiotherapy in high-risk populations, as with patients with traumatic brain injury. Surgery is the last resort, specifically in cases of intractable pain, impingement of important neurovascular structures, and pressure ulcers and to improve mobility. The timing of surgical intervention remains controversial; orthopedic surgeons recommend earlier resections to improve mobility, and neurosurgeons recommend later resection to allow bone maturation and normalization of alkaline phosphatase levels to reduce recurrence. During operative resection, ketamine infiltration in the wound bed can help suppress inflammatory cascades that can aggravate postoperative pain and reduce recurrence rate without affecting local wound healing.

A better understanding of the pathophysiologic process of heterotopic calcification can lead to better prevention and treatment in the future.
